# Paradoxical systemic manifestations from right-sided infective endocarditis via a transient intrapulmonary shunt: a case report

**DOI:** 10.1093/ehjcr/ytag481

**Published:** 2026-06-23

**Authors:** Yoshihito Saijo, Hirotsugu Yamada, Eriko Nasu, Shusuke Yagi, Masataka Sata

**Affiliations:** Department of Cardiovascular Medicine, Tokushima University Hospital, 2-50-1, Kuramoto-cho, Tokushima 770-8503, Japan; Department of Cardiovascular Medicine, Tokushima University Hospital, 2-50-1, Kuramoto-cho, Tokushima 770-8503, Japan; Department of Cardiovascular Medicine, Tokushima University Hospital, 2-50-1, Kuramoto-cho, Tokushima 770-8503, Japan; Department of Cardiovascular Medicine, Tokushima University Hospital, 2-50-1, Kuramoto-cho, Tokushima 770-8503, Japan; Department of Cardiovascular Medicine, Tokushima University Hospital, 2-50-1, Kuramoto-cho, Tokushima 770-8503, Japan

**Keywords:** Right-sided infective endocarditis, Tricuspid valve, Septic pulmonary embolism, Paradoxical embolism, Echocardiography, Microbubble, Agitated-saline contrast, Case report

## Abstract

**Background:**

Right-sided infective endocarditis (RSIE) commonly causes septic pulmonary embolism, whereas systemic manifestations typically suggest concomitant left-sided infective endocarditis or an intracardiac right-to-left shunt such as patent foramen ovale (PFO).

**Case summary:**

A 24-year-old man with atopic dermatitis presented with fever and painful distal extremity skin lesions. Three sets of blood cultures grew *Staphylococcus aureus*. Transthoracic echocardiography (TTE) revealed a highly mobile, club-shaped vegetation (18.6 × 10 mm) adjacent to the tricuspid valve with severe tricuspid regurgitation due to leaflet perforation. Agitated-saline contrast TTE demonstrated right-to-left shunting with Grade III microbubble appearance in the left heart after five cardiac cycles following release of Valsalva manoeuvre, whereas no microbubbles were detected in the left heart at rest. Transoesophageal echocardiography confirmed no PFO and no left-sided vegetation. Chest computed tomography (CT) demonstrated multiple pneumonia and lung abscesses with cavities, consistent with septic pulmonary embolic disease, and discitis was confirmed on magnetic resonance imaging. Skin biopsy of the distal extremity lesions was consistent with septic micro-embolization. Empirical intravenous antibiotics were initiated and then tailored to susceptibility results. Antibiotic therapy continued for 6 weeks from the first documented negative blood culture. The vegetation regressed without surgery and pulmonary lesions improved. At 12-month follow-up, chest CT showed improvement of lung complications, and repeat agitated-saline contrast TTE suggested disappearance of right-to-left shunting.

**Discussion:**

This case represents a plausible mechanism of paradoxical systemic manifestations in RSIE via a transient intrapulmonary shunt during severe septic pulmonary embolic disease.

Learning points

- Systemic manifestations in right-sided infective endocarditis should prompt careful assessment–evaluation to detect a cause of right-to-left shunt.
- Transient intrapulmonary shunts may provide a pathway for paradoxical systemic manifestations. Agitated-saline contrast echocardiography supports this mechanism.

## Introduction

Right-sided infective endocarditis (RSIE) typically causes septic pulmonary emboli and pulmonary complications including pneumonia and lung abscesses.^1^ In contrast, systemic manifestations are uncommon in isolated RSIE and usually indicate either concomitant left-sided endocarditis or the presence of the right-to-left shunt like a patent foramen ovale (PFO).^2,3^ We report a case of isolated RSIE with paradoxical systemic manifestations, where detailed echocardiographic study excluded the presence of PFO and left-sided vegetations.

## Summary figure

Timeline of clinical course

**Figure:**
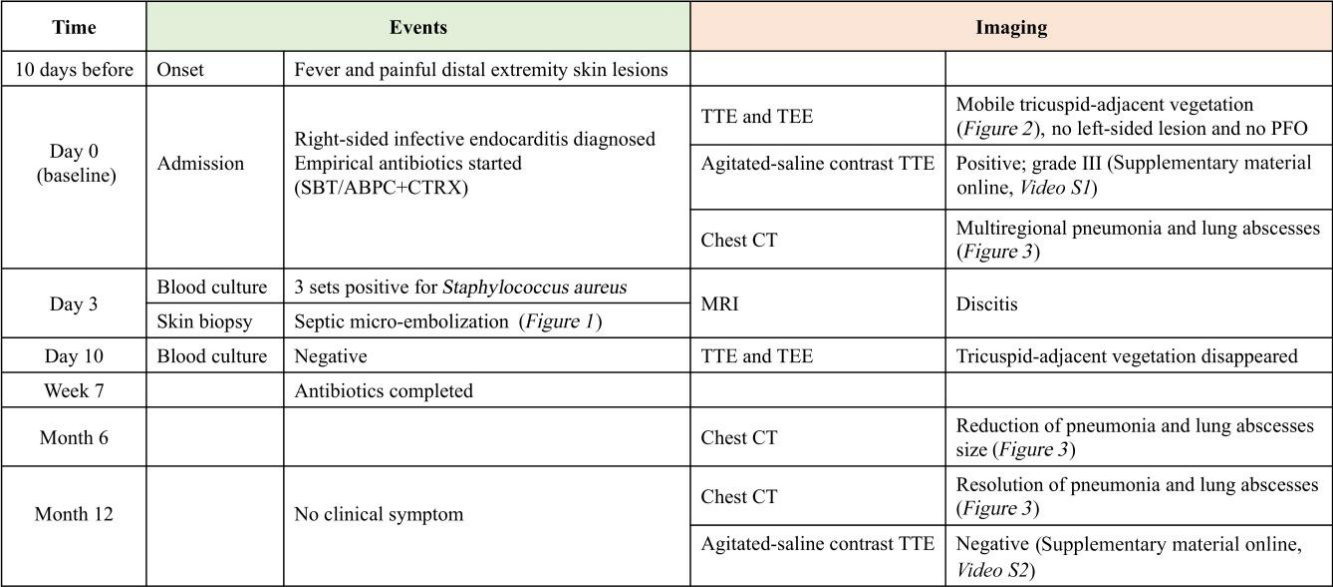


## Case presentation

**Figure 1:**
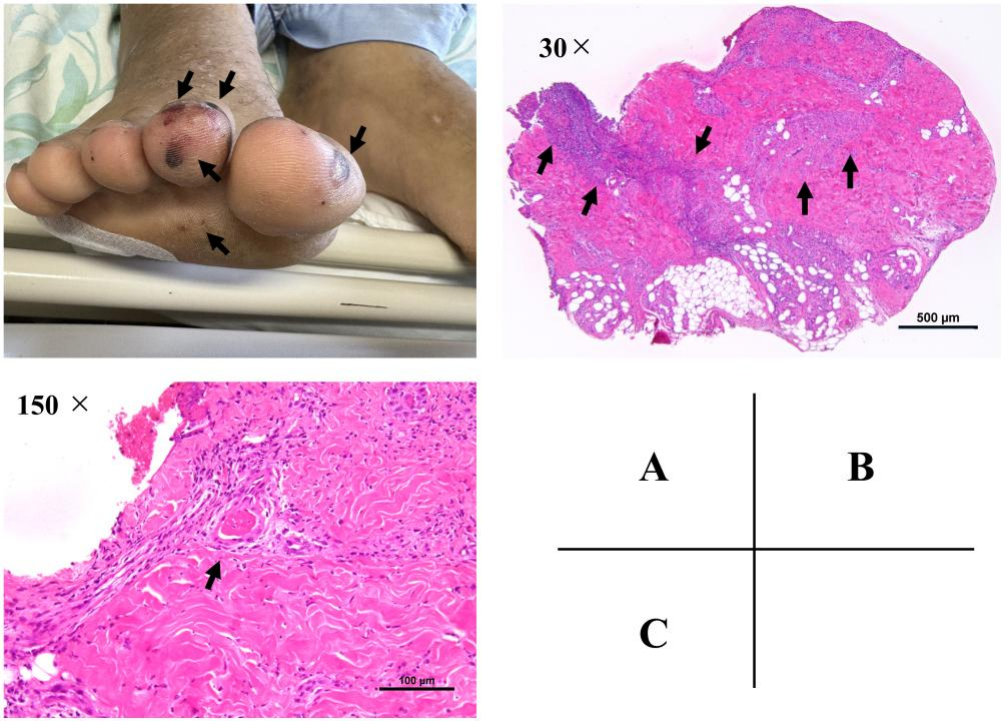
Clinical photography shows painful skin lesions (arrows) at the distal toes of the right foot (*A*). Histopathology demonstrates predominantly neutrophilic inflammatory cell infiltration in the dermis with focal areas of necrosis and microabscess formation (arrows) (*B*). A dermal vessel containing an intraluminal thrombus with surrounding inflammatory cell infiltration (arrows) (*C*).

**Figure 2:**
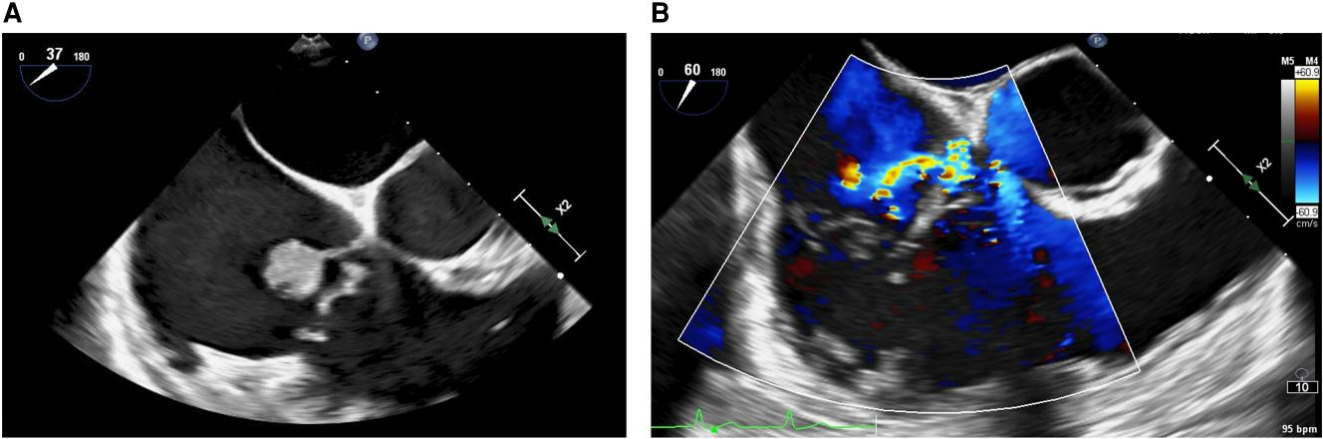
Transoesophageal echocardiography demonstrated a highly mobile, club-shaped vegetation (18.6 × 10 mm) adjacent to the tricuspid valve (*A*). Severe tricuspid regurgitation due to septal leaflet rupture was observed (*B*). Transoesophageal echocardiography also confirmed no patent foramen ovale on colour Doppler imaging and no left heart-sided vegetation.

**Figure 3:**
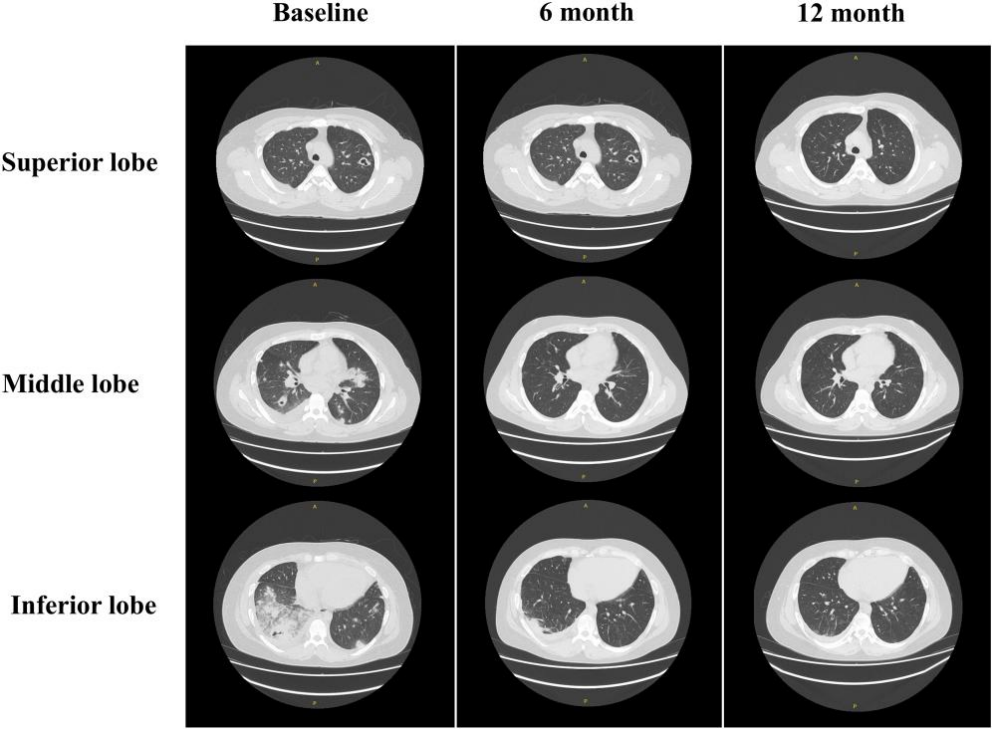
Chest computed tomography at baseline demonstrated multifocal pneumonia and lung abscesses, consistent with septic pulmonary embolic disease. Serial follow-up computed tomography showed improvement in lung involvement after antibiotic therapy for infective endocarditis.

A 24-year-old man with atopic dermatitis presented with 10 days of fever and painful distal extremity skin lesions. On admission, blood pressure was 126/79 mmHg, heart rate 82 b.p.m., oxygen saturation 99% (room air), and body temperature 37.4°C. Physical examination revealed painful skin lesions at the right toes (*Figure 1A*) and back pain over the lumbar spine. There were no focal neurological deficits. Laboratory tests showed a white blood cell count of 23.0 × 10^3^/μL and C-reactive protein of 23.2 mg/dL. Transthoracic echocardiography (TTE) demonstrated a highly mobile, club-shaped vegetation (18.6 × 10 mm) adjacent to the tricuspid valve with severe tricuspid regurgitation due to leaflet perforation (*Figure 2*). Right ventricular systolic function was preserved [tricuspid annular plane systolic excursion (TAPSE) 20 mm] at baseline. Agitated-saline contrast TTE demonstrated right-to-left shunting with Grade III microbubble appearance in the left heart after five cardiac cycles following release of the Valsalva manoeuvre, whereas no microbubbles were detected in the left heart at rest (see Supplementary material online, *Video S1*). Given the delayed timing of left heart opacification, an intracardiac shunt such as PFO was considered unlikely, and an intrapulmonary shunt mechanism was favoured. Transoesophageal echocardiography (TEE) also confirmed the right-sided vegetation and demonstrated no PFO on colour Doppler imaging and no left-sided vegetations. Chest computed tomography (CT) revealed multifocal pneumonia and lung abscesses with cavity compatible with septic pulmonary embolic disease (*Figure 3*). Contrast-enhanced CT showed no other extracardiac shunts, and discitis was confirmed on magnetic resonance imaging. Empirical intravenous antibiotics (sulbactam/ampicillin and ceftriaxone) were initiated and later tailored according to susceptibility testing (continued sulbactam/ampicillin). Three sets of blood cultures obtained on admission grew *Staphylococcus aureus* on Day 3. Histopathology of distal toe lesions demonstrated predominantly neutrophilic perivascular inflammation with focal dermal necrosis and microabscess formation, and an eosinophilic intraluminal structure was identified in a dermal vessel, suggestive with septic micro-embolization (*Figure 1B* and *C*). Blood cultures became negative on Day 10, and intravenous antibiotics were continued for 6 weeks from the first documented negative blood culture. On therapeutic process, the vegetation regressed on serial echocardiography, and surgery was deferred. Follow-up chest CT at 6 months showed regression of pulmonary complications, and at 12 months showed resolution. Then, repeat agitated-saline contrast TTE demonstrated disappearance of right-to-left shunting (see Supplementary material online, *Video S2*). Right ventricular systolic function remained stable at follow-up (TAPSE 19 mm).

## Discussion

Systemic manifestations in isolated RSIE are unusual and most often explained by concomitant presence of left-sided infective endocarditis or an intracardiac right-to-left shunt.^4^ In the present case, careful examinations excluded both PFO and left-sided vegetations. Notably, agitated-saline contrast TTE showed delayed microbubble appearance in the left heart, supporting transpulmonary passage rather than intracardiac shunting.^5,6^ Chest CT demonstrated multifocal pneumonia and lung abscesses consistent with septic pulmonary embolic disease. These findings suggest that an intrapulmonary right-to-left shunt may provide a pathway for paradoxical systemic manifestations via lung involvement from septic pulmonary embolism in patients with isolated RSIE.

We propose that severe septic pulmonary embolic disease with multifocal pneumonia and lung abscesses was associated with transient intrapulmonary right-to-left shunt physiology, allowing paradoxical passage of embolic material from the right heart into the systemic circulation. Chronic infection has been reported as an uncommon cause of acquired pulmonary arteriovenous malformations.^7,8^ Moreover, case literature has suggested that recurrent endocarditis can be associated with pulmonary vascular complications culminating in the formation of pulmonary arteriovenous malformations, supporting the biological plausibility of infection-related arteriovenous communications.^9^ Pulmonary arteriovenous malformations create a continuous anatomic right-to-left shunt between pulmonary arteries and veins, and they are well recognized as a cause of paradoxical embolic complications.^10–12^ Therefore, once a pulmonary right-to-left shunt is present, both thrombotic and septic embolic material from the RSIE may reach the systemic circulation. No structural pulmonary arteriovenous malformations or other extracardiac shunt was identified on contrast-enhanced CT. Although tiny intermittent intracardiac shunts cannot be completely excluded and should be acknowledged as a limitation, the delayed microbubble appearance pattern in the left heart is consistent with an intrapulmonary shunt mechanism .^9^ Accordingly, an intrapulmonary right-to-left shunt was considered as the most plausible route for paradoxical systemic manifestations in this case.

A key supportive feature for hypothesis was reversibility. In the present case, the right-to-left shunt disappeared after infection control and improvement of lung involvement, arguing against a fixed congenital shunt such as a congenital microscopic arteriovenous malformation. Therefore, we speculate that the right-to-left shunt was functional and reversible rather than a permanent anatomical pulmonary arteriovenous malformation. In severe pulmonary infection and sepsis, pulmonary vascular dysregulation, such as impaired hypoxic pulmonary vasoconstriction and inflammation-driven angiogenic signalling (e.g. vascular endothelial growth factor/hypoxia-inducible factor), may further increase perfusion of poorly ventilated regions, and intrapulmonary arteriovenous anastomoses can be recruited under hypoxia and stress-related conditions.^13,14^ Although the mechanisms of transient shunt recruitment in septic pulmonary embolic disease remain incompletely understood, the temporal relationship between improvement of pulmonary complications and disappearance of shunt supports a functional intrapulmonary mechanism.

## Conclusion

A transient intrapulmonary right-to-left shunt associated with multiple septic pulmonary embolic disease may provide a plausible pathway for systemic manifestations in isolated RSIE. Serial agitated-saline contrast echocardiography and serial chest CT may support this mechanism by demonstrating reversibility of both shunt physiology and pulmonary lesions.

## Supplementary Material

ytag481_Supplementary_Data

## Data Availability

The data underlying this article are available in the article on reasonable request.

## Lead author biography

Dr Yoshihito Saijo graduated from the Tokyo Medical University in Tokyo, Japan, in 2011. He completed his speciality in cardiovascular medicine at the faculty of Tokushima University Hospital and is currently a cardiologist at the same institution. Areas of interest included heart failure, valvular disease, cardiotoxicity, and cardiomyopathy.

## Notes

**Consent:** The authors confirm that written consent for submission and publication of this case report, including imaging and associated text, has been obtained from the patient in line with COPE guidance.
